# Supporting and Incentivizing Peer Leaders for an Internet-Based Private Peer Community for Youths With Type 1 Diabetes: Social Network and Directed Content Analysis

**DOI:** 10.2196/48267

**Published:** 2023-12-12

**Authors:** Nancy Wu, Susan Joanne Wang, Anne-Sophie Brazeau, Deborah Chan, Joseph Mussa, Meranda Nakhla, Mariam Elkeraby, Maryna Ell, Melinda Prevost, Laurie Lepine, Constadina Panagiotopoulos, Geetha Mukerji, Sonia Butalia, Mélanie Henderson, Deborah Da Costa, Elham Rahme, Kaberi Dasgupta

**Affiliations:** 1 Centre for Outcomes Research and Evaluation Research Institute of the McGill University Health Centre Montreal, QC Canada; 2 School of Human Nutrition Faculty of Agricultural and Environmental Sciences McGill University Ste Anne de Bellevue, QC Canada; 3 Patient partner Montreal, QC Canada; 4 Endocrinology & Diabetes Unit, British Columbia Children's Hospital and Department of Pediatrics University of British Columbia Vancouver, BC Canada; 5 Department of Pediatrics University of British Columbia Vancouver, BC Canada; 6 Institute of Health Policy, Management and Evaluation Health Sciences Building University of Toronto Toronto, ON Canada; 7 Department of Medicine, Cumming School of Medicine Health Sciences Centre Foothills Campus, University of Calgary Calgary, AB Canada; 8 Department of Community Health Sciences, Cumming School of Medicine University of Calgary Calgary, AB Canada; 9 Department of Pediatrics Université de Montréal Montreal, QC Canada; 10 Centre de Recherche CHU Sainte-Justine Montreal, QC Canada; 11 School of Public Health, Department of Social and Preventive Medicine Université de Montréal Montreal, QC Canada; 12 Department of Medicine McGill University Health Centre Montreal, QC Canada

**Keywords:** Facebook, chronic disease communities, internet-based communities, type 1 diabetes, adolescents, young adults, peer support

## Abstract

**Background:**

Youths with type 1 diabetes (T1D) frequently experience stigma. Internet-based peer communities can mitigate this through social support but require leaders to catalyze exchange. Whether nurturing potential leaders translates into a central role has not been well studied. Another issue understudied in such communities is lurking, the viewing of exchanges without commenting or posting.

**Objective:**

We aimed to assess the centrality of the peer leaders we selected, trained, and incentivized within the Canadian Virtual Peer Network (VPN)-T1D. This is a private Facebook (Meta Platforms, Inc) group that we created for persons aged 14 to 24 years with T1D. We specifically sought to (1) compare a quantitative estimate of network centrality between peer leaders and regular members, (2) assess the proportions of network exchanges that were social support oriented, and (3) assess proportions of high engagement (posts, comments, reactions, and votes) and low engagement (lurking) exchanges.

**Methods:**

We recruited peer leaders and members with T1D from prior study cohorts and clinics. We trained 10 leaders, provided them with a monthly stipend, and encouraged them to post on the private Facebook group we launched on June 21, 2017. We extracted all communications (posts, messages, reactions, polls, votes, and views) that occurred until March 20, 2020. We calculated each member’s centrality (80% of higher engagement communications comprising posts, comments, and reactions plus 20% of members with whom they connected). We divided each member’s centrality by the highest centrality to compute the relative centrality, and compared the mean values between leaders and members (linear regression). We calculated the proportions of communications that were posts, comments, reactions, and views without reaction. We performed content analysis with a social support framework (informational, emotional, esteem-related, network, and tangible support), applying a maximum of 3 codes per communication.

**Results:**

VPN-T1D gained 212 regular members and 10 peer leaders over 33 months; of these 222 members, 26 (11.7%) exited. Peer leaders had 10-fold higher relative centrality than regular members (mean 0.53, SD 0.26 vs mean 0.04, SD 0.05; 0.49 difference; 95% CI 0.44-0.53). Overall, 91.4% (203/222) of the members connected at least once through posts, comments, or reactions. Among the 75,051 communications, there were 5109 (6.81%) posts, comments, and polls, 6233 (8.31%) reactions, and 63,709 (84.9%) views (lurking). Moreover, 54.9% (3430/6253) of codes applied were social support related, 66.4% (2277/3430) of which were informational (eg, insurance and travel preparation), and 20.4% (699/3430) of which were esteem related (eg, relieving blame).

**Conclusions:**

Designating, training, and incentivizing peer leaders may stimulate content exchange and creation. Social support was a key VPN-T1D deliverable. Although lurking accounted for a high proportion of the overall activity, even those demonstrating this type of passive participation likely derived benefits, given that the network exit rate was low.

**International Registered Report Identifier (IRRID):**

RR2-10.2196/18714

## Introduction

### Background

Type 1 diabetes (T1D) is a lifelong chronic condition characterized by complex self-management [1]. It requires the self-administration of insulin through injections or continuous infusion. It necessitates the self-monitoring of blood sugar (glucose) through readers (glucometers) and finger pricks, or continuous glucose monitors inserted into the skin. Physical activity, food intake, and insulin must all be considered to prevent both high glucose levels, which can lead to blindness [2], kidney injury [2], heart disease [3], and stroke [3], and very low glucose levels, which can cause confusion, coma, and death [4,5].

A large proportion of people with this condition experience onset during childhood, adolescence, or young adulthood [6]. In a previous Canadian internet-based study that we conducted [7], 65% of youths aged 14 to 24 years with T1D reported stigma related to the disease. They indicated the avoidance of disclosing their diagnosis or managing the disease in front of either friends or strangers. Many people living with T1D do not have friends or acquaintances with this condition [8]. To provide a forum for peer relatedness and social support, we built the Canadian Virtual Peer Network (VPN)-T1D, a private Facebook (Meta Platforms, Inc) group [9]. As reviewed by Hilliard and colleagues [10], many T1D forums and communities have formed organically as sources of social support with the emergence of internet-based communications. We adopted a more constructed approach, recruiting peer leaders and regular members from stigma study participants [7], clinics, and social media. With funding through a patient-oriented research grant from the Canadian Institutes of Health Research and partnership funding from Diabetes Canada, we provided leaders with a monthly stipend to stimulate discussion, and we organized a 1-day training workshop, covering expenses.

With the overarching goal of iterative enhancement, we sought to better understand the network’s structure, dynamics, and leadership, and to evaluate its relevance to members. Specifically, we aimed to (1) evaluate whether our selected, trained, and incentivized peer leaders did play a measurably central role; (2) assess whether network communications were social support oriented; (3) ascertain the proportions of different types of exchanges, including, importantly, the more passive lurking (views without reaction); and (4) delineate the topics of importance to members. To achieve these aims, we drew on a combination of social network analysis (SNA), linear regression, and directed content and thematic analyses.

SNA conceptualizes and illustrates members as *nodes,* and connections between them as *edges* [11,12]. Christakis and Fowler [13] applied SNA methods to illustrate connections between Framingham cohort members (friends, partners, neighbors, and family members) and demonstrated that connections to members who gained excess weight predicted gaining excess weight. Edwards and colleagues [14] applied these methods to identify popular opinion leaders for a youth-led sexual violence prevention initiative. These researchers asked participants about relationships in real-world settings (eg, friends, family, and perceived leaders), but such methods are also applied to internet-based communities.

Furthermore, 3 papers from 2012 to 2013, 1 by Dias and colleagues [15] and 2 by Chomutare and colleagues [16,17], report the use of SNA methods in internet-based diabetes forums, representing members as nodes and communications between them as edges. Their focus was on the delineation of communities within diabetes forums using greedy optimization (Girvan-Newman algorithm and hierarchical agglomeration). One of the papers examined a single diabetes forum and indicated that a longer diabetes duration was associated with being a central network member [15]. Another study evaluated 5 forums, showing that 6 (60%) out of 10 central members overlapped across the 5 forums [16]. The third paper evaluated social networks within 2 diabetes forums over 4 to 6 years, documenting the dynamic nature of communications and connections among the members, and demonstrating that the subcommunities that they identified engaged for short periods over time [17].

### This Study

In contrast to these studies, we applied SNA methods within a defined youth T1D community that did not form organically within a forum but that we purposefully created through a partnership between researchers, health professionals, and persons living with T1D. We wanted to see whether cultivating a group of individuals as leaders (by selecting, training, and incentivizing through a monthly stipend) would translate into these individuals actually emerging as leaders, as captured by the SNA construct of centrality.

Many evaluations of internet-based diabetes communities involve thematic analyses and enumeration of posts, comments, and reactions [18-27]. We conducted both thematic analyses of communications and a rubric-guided directed content analysis to determine the proportion of social support–related (informational, emotional, esteem-related, network, and tangible support) communications, given that social support was a key goal of VPN-T1D creation. We enumerated posts, comments, and reactions, as well as views without comments or reactions. Individuals who participate exclusively through such views are termed *lurkers*. There has been a limited number of studies on lurkers, as only Facebook page administrators have access to this metric, but a 2020 examination of changes in lurker status within an internet-based diabetes community suggested that 90% of members are lurkers, consistent with other types of internet-based communities [28-30].

We believe that our multimethod approach resulted in a sophisticated portrait of a chronic disease network, which is a launching point for further enhancements relevant across internet-based chronic disease peer communities.

## Methods

### Ethical Considerations

We received ethics approval from the McGill University Health Centre Research Ethics Board on May 20, 2020 (MP-37-2020-6511), which did not require us to seek individual written informed consent. Instead, as described in our published protocol [9], we provided a reader-friendly description of the study with an invitation for members to comment. We did not receive any specific comments. All data were deidentified before analysis, with the replacement of names by study numbers. We did not provide any form of compensation or monetary incentive to regular network members. We did provide designated peer leaders with a monthly stipend, as discussed in the Peer Leaders subsubsection under the Sample Population and Recruitment subsection. We conducted SNA, directed content analysis, and thematic analysis of VPN-T1D private Facebook exchanges between June 21, 2017, and March 20, 2020.

### Sample Population and Recruitment

The recruitment of both peer leaders and regular members began in June 2017, with recruitment and training of peer leaders between June and November 2017, followed by recruitment of regular members on a continuous basis.

#### Peer Leaders

We reviewed free-text narratives from our previous stigma study [7] and, based on these, invited 43 respondents to become leaders, among whom 5 (12%) volunteered. We recruited 5 patients whom we believed to be suitable peer leaders from our clinics. From each of these sources, 2 patients declined before launch (2/10, 20%). We recruited 2 more patients through DSkate, an organization that assists children and youths with T1D in playing hockey safely. We invited leaders to Edmonton, Canada, for the annual 2017 Diabetes Canada Conference and a half-day training session [31] (with expenses paid; 7 attended training in person, 1 via teleconference, and 2 received audio recordings of the training). We maintained regular contact and provided a monthly stipend of CAD $50 (US $36.38) from December 2017 to April 2019 and CAD $25 (US $18.19) subsequently. A team member or delegate reviewed all the communications posted to ensure courteous exchanges.

#### Recruitment of Regular Members

We invited stigma study participants to join VPN-T1D, collaborated with diabetes organizations on social media recruitment posts, and placed posters and flyers at clinics, with intermittent reminders to clinic staff. Candidates made a join request via secure website or email for eligibility verification (ie, 14 to 24 years of age and presence of T1D); we also collected data on the age at diagnosis, use of insulin pumps versus insulin injections, and town of residence.

### Network Structure, Dynamics, and Individual Member Centrality

#### Baseline Characteristics

We enumerated the total number of members who joined the group and the number of members who remained at the end of the observation period. We computed frequencies, along with proportions, for the region of residence, sex, the age at diagnosis (<14 years, 14 years, or >14 years), and the use of insulin pumps versus insulin injections. We did not query the last 2 variables among leaders. We inferred sex from names (female-sounding, male-sounding, or unclear), acknowledging this as a crude method.

#### Stable Membership

We calculated the monthly average of new members. Using a Poisson distribution with this average as the mean, we estimated 6 to be the lowest number of new members, for which there was a <50% probability of occurrence in any month. We defined the beginning of each month with ≥6 new members as the start of a new period.

#### Communication Types

We manually abstracted communications and classified each as a post, comment, reaction, poll, vote, or view. As in an SNA of a Facebook group focused on smoking cessation, we considered posts and comments to be high engagement, reactions to be medium engagement, and views to be low engagement [32].

### SNA Method

We applied SNA, a method rooted in sociology, with defined parameters that reflect the social dynamics of a network’s members [12].

#### Network Visualization

##### Overview

Each network member was depicted as a node (dot), and each node pair was connected by a line if there were ≥1 high or medium engagement exchanges between the members (*sna* in R [version 3.5.1; R Core Team] and *network* in Rstudio [version 1.1.456; Rstudio, Inc]). The software placed the members who shared more connections closer together. We constructed visualizations for the full observation period and for each stable membership period, arranging the latter sequentially into a video.

##### Density and Cliques

*Density*, defined as the number of node pairs with at least 1 moderate to high engagement interaction divided by the total number of possible node pairs, was calculated for each stable membership period. *Cliques* were node groupings with a high or medium engagement interaction between all possible pairs within them; we calculated the number and size of cliques for the full observation period.

##### Relative Centrality

We defined *centrality* for each member, for each stable time interval, as (1) 80% of the total number of their high and medium engagement connections plus (2) 20% of the total number of members with whom they connected through high and medium engagement exchanges. We divided this value by the highest centrality within the interval considered (*relative centrality*). We computed the mean (SD) relative centrality for each member, across intervals. We computed the group means (SDs) for peer leaders and regular members. For peer leaders, we considered only the periods in which they remained peer leaders. In a linear regression model, we examined the association between designated peer leader status and relative centrality. We considered sex and age at T1D diagnosis for inclusion in the model (univariate association with relative centrality *P*≤.25 and multivariable association *P*≤.05).

### Directed Content Analysis

Using a rubric developed for a private Facebook group of an HIV program [33], 2 research team members classified each post and comment as *seeking support*, *providing support*, or *neither*. We further classified these posts and comments related to social support as *informational* (helping solve a problem), *emotional* (supporting emotional expression and providing a sense of warmth), *esteem-related* (validating self-concept or rights), *network* (expanding structural connections), and *tangible* (providing or offering specific materials or services) support. Within these subcategories, there was a third level of classification [33] (*informational support*: advice, referral, situational appraisal, teaching, and other informational support; *emotional support*: relationship, physical affection, confidentiality, sympathy, empathy, encouragement, prayer, and other emotional support; *esteem-related support*: compliment, validation, relief of blame, and other esteem-related support; *network*: access, presence, companionship, and other network; *tangible support*: loan, perform direct task, perform indirect task, active participation, express willingness, and other tangible support).

We created separate codes for other types of exchanges: socializing, conversation starters or contributors, administrative, expressions of gratitude, banter and memes, research, offering congratulations, group cohesion, negative interaction, community protection, nonverbal cues, and miscellaneous. We allowed up to 3 codes per communication from the third-level social support categories and from among the codes for other types of exchanges that we created. Two team members classified the exchanges (NW and SJW) with some assistance from a third member (JM), and a fourth member advised for the resolution of coding differences (KD).

### Thematic Analysis for Ascertaining General Themes and Priorities

Several research team members (NW, SJW, DDC, ASB, MN, MH, CP, SB, GM, and KD) participated in a separate review of the categorized communications to identify overarching themes. At least 3 research team members independently reviewed the exchanges within each second-level category and proposed theme labels. Thereafter, 3 research team members (NW, SJW, and KD) distilled these into 10 final themes.

## Results

### Baseline Characteristics

Over 33 months, in addition to the 10 peer leaders, 212 regular members joined the group. The 222 leader and regular members included 70.3% (n=156) members with a female-sounding name, 25.2% (n=56) members with a male-sounding name, and 4.5% (n=10) members for whom the name was nonspecific. More than half (127/222, 57.2%) of the members resided in Central Canada, 13.5% (30/222) of the members resided in the Prairie provinces, 10.8% (24/222) of the members resided in Atlantic Canada, 7.7% (17/222) of the members resided in British Columbia, and 10.8% (24/222) of the members did not specify where they resided. Among the 212 regular members, 66.5% (n=141) were aged <14 years at diagnosis, 11.8% (n=25) were older, and 21.7% (n=46) did not provide this information. Approximately half (112/212, 52.8%) of the regular members used insulin pumps, just over one-quarter (56/212, 26.4%) reported multiple injections, and 20.8% (44/212) did not specify.

### Continued Membership and Stable Periods

Of the 222 members, there were 196 (88.3%) on March 20, 2020, indicating that 26 (11.7%) had exited the group. The 10 leaders continued in their roles for a median of 32 months. Specifically, 6 (60%) of the 10 leaders continued beyond March 20, 2020; 1 (10%) turned 24 years of age and exited; and 3 (30%) indicated that they were too busy to continue. We identified 13 stable membership periods (median 1 [range 1-10] month).

### Communications and Network Visualizations

**Figure 1:**
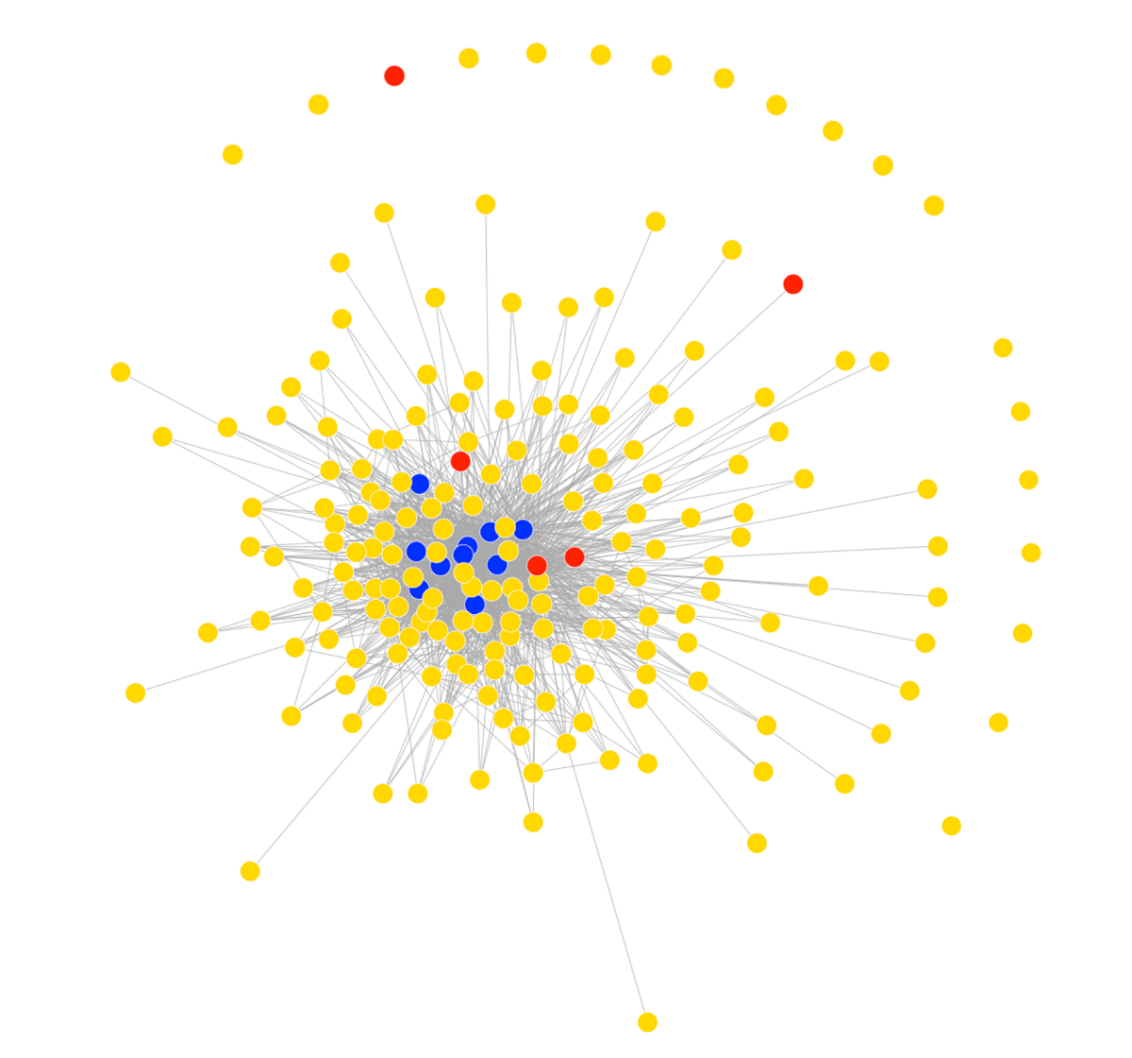
Network visualization for the 33-month observation period. Each colored circle represents a node or individual network member (yellow=regular member; blue=designated peer leader; and red=administrator). The line connecting node pairs indicates that a medium to high engagement exchange occurred ≥1 times between the corresponding members. The nodes representing members with more connections are spaced closer together.

There were a mean of 33 (SD 19) posts and polls per month. The ratio of comments, reactions, and views to posts were 3.6 to 1, 5.3 to 1, and 57 to 1, respectively; the ratio of votes to polls was 27 to 1. The 75,051 communications abstracted included 1096 (1.46%) posts, 3998 (5.33%) comments, 5835 (7.77%) reactions, 15 (0.02%) polls, 398 (0.53%) poll votes, and 63,709 (84.89%) views. Across time, of the 222 members, 203 (91.4%) connected at least once through a moderate or high engagement communication. In the first month (Video S1 in Multimedia Appendix 1), membership mostly included peer leaders and administrators. Over time, regular members joined, and more connections formed. In Figure 1, the peer leaders and regular members who shared more connections are spatially closer to one another, whereas those who did not participate in any high-medium engagement interactions are unconnected and on the periphery of the visualization, as views are not depicted by lines.

### Network Growth, Density, and Cliques

**Figure 2:**
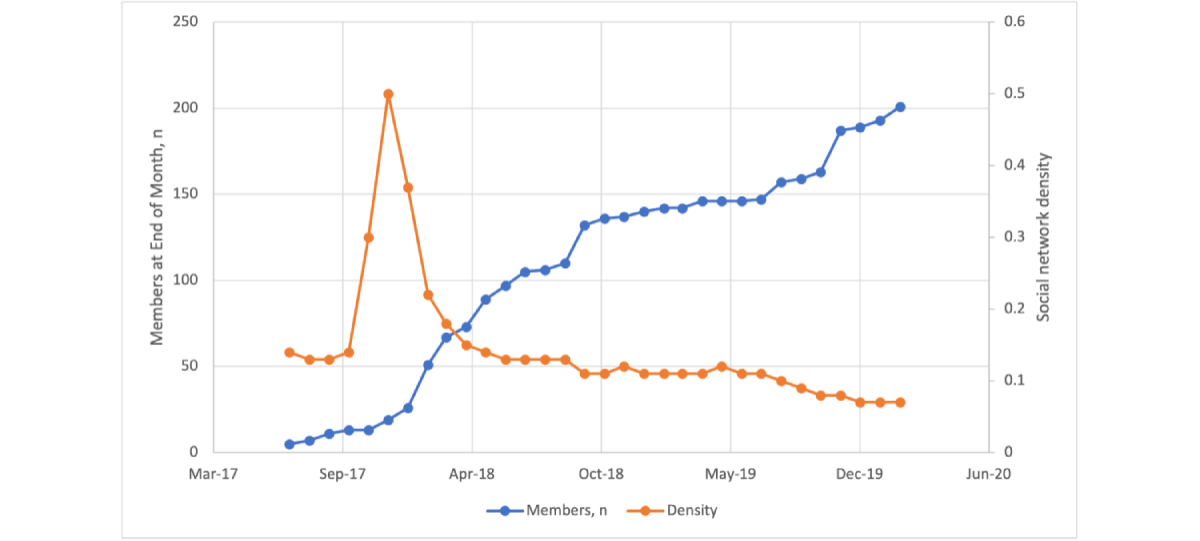
Number of members (blue) and network density (orange) at the end of each month, from July 2017 to February 2020.

**Figure 3:**
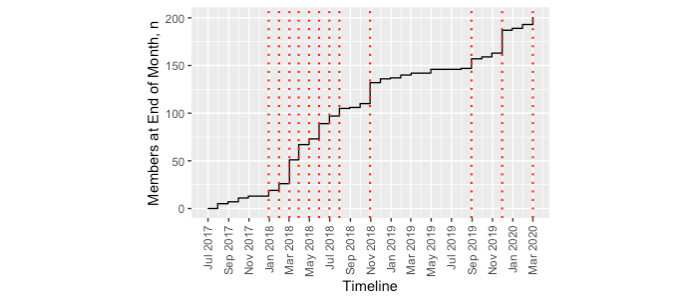
Number of members at the end of each month, from July 2017 to February 2020. Vertical dotted lines delineate the start or end of a stable time interval. The longest stable time interval is November 2018 to September 2019.

There were fewer members and they interacted more intensively with one another, reflected by a peak density of 0.5 (Figures 2 and 3) at the outset and lower values later in in the development of the network. The density declined below 0.4 at approximately 50 members, and after approximately 70 members, the density remained around 0.1 (Figure 2). The mean density was 0.071 (SD 0.09). There were 5 cliques, each including 17 participants and all including 8 peer leaders and the main VPN-T1D administrative account.

### Relative Centrality

**Figure 4:**
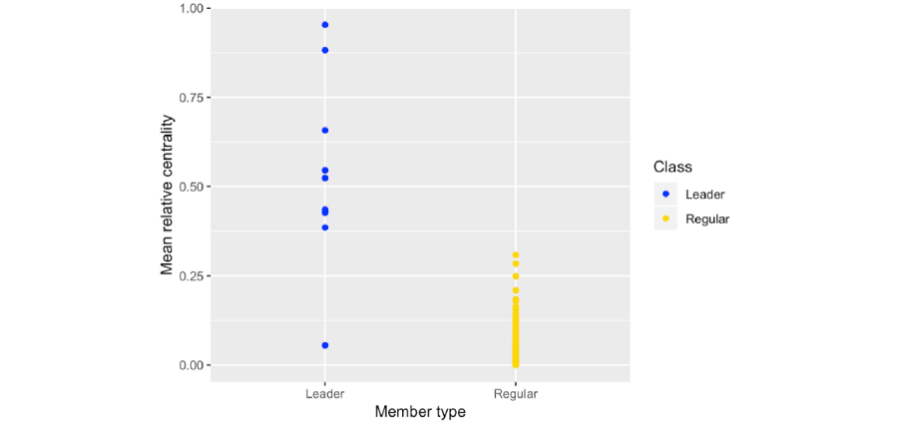
Mean relative centrality of the designated peer leaders and regular members.

Individual mean relative centrality values (Figure 4) across the periods of stable membership were 0.53 (SD 0.26) for peer leaders and 0.04 (SD 0.05) for regular members. In a linear regression model, peer leader status was associated with a 0.49 higher relative centrality value (95% CI 0.44-0.53). Age at T1D diagnosis and sex inferred from name were not associated with relative centrality.

### Frequency and Types of Social Support

Communications were courteous. Across 5450 posts, polls, and comments, we applied 6253 codes. Overall, 45.15% (2823/6253) of codes were related to forms of socializing that did not formally fit into the social support categories (Table S1 in Multimedia Appendix 2), and 54.9% (3430/6253) of codes were social support related. Of these, 67.1% (2303/3430) were offering support and 32.9% (1127/3430) were requesting or receiving support. In terms of types of support, more than half (2277/3430, 66.4%) were informational support, addressing topics such as bleeding at the site of sensor placement, environment-friendly disposal of supplies, travel preparation related to diabetes, and insurance challenges (Textbox 1). Approximately one-fifth (699/3430, 20.4%) of them were esteem related, such as relieving blame by acknowledging self-management challenges, celebrating members who rose to such challenges, and affirming expressions of feelings related to diabetes. Emotional, network, and tangible support were less prominent, although there was arguably some overlap between the esteem and emotional categories.

**Frequency and examples of text coded as social support.**

**Informational support (2277/3430 comments, 66.38%)**

- Advice
  “I think my pump site is in muscle...which is kinda nice because that used to be fat but kinda not nice because it hurts and I’m worrying. Have you ever done this?”
  “This happens to me when I use my legs—just keep an eye on it, I usually rip mine if they start to ache at all” (response to the previous comment).
  “Hey guys I’m going to the Philippines for 20 days and I don’t really know how to prepare myself. What important things should I know when I’m traveling”
  “Take all supplies on you on the plane...just in case your baggage gets lost” (response to the previous comment).
- Referral
  “...Went to pick up some prescriptions after my appointment and realized that my insurance is finally expired (I’m out of school this year for the first time so I’m no longer covered under my dad’s insurance policy).... I was really shocked and upset by this. Can anyone guide me towards some funding/financial assistance that would help me to get some of this covered in some way?”
  “[Company name] has a ‘Severe hypo[glycemia] rescue plan’ printout that you might find useful: [link]. Have you talked to your friends & fam about hypo[glycemia episode]s?”
- Situational appraisal
  “I never looked at it like that, like that we act as our pancreas is supposed to. I think that’s really cool and I’m going to use that.”
  “I’ve never had that before. But maybe there is something else that has changed that’s overriding the normal high blood glucose.”
- Teaching
  “Hi! I just put in a new libre, but when I pulled the big part off (once inserted), it squirted blood, and a bunch of blood was coming out of the centre part. I’m just wondering if it’s still usable.”
  “I had a nurse at camp tell me once that ‘bleeders are readers’ with CGM/FGMs [continuous glucose monitoring/flash glucose monitoring]!” (response to the previous comment)
  “This may be an odd/dumb question, but are test strips recyclable?? I feel awful with the amount of waste I have with all my diabetes supplies!”
  “I hate the waste but I don’t think they’re recyclable. The blood probably negates them, and they’re technically electronics (if you peel them apart you can see they’re little microchips)” (response to the previous comment).
- Other informational support
  “Which types of insulin are you using and when?”

**Esteem-related support (699/3430, 20·38%)**

- Compliment
  “That is so cool that you noticed that detail! You must be so perceptive.”
  “Super proud of you, (tagged name)! Easier said than done, but never let the pod (or diabetes for that matter) bring you down! Keep doin you.”
- Validation
  “Anyone else feel that having high blood sugar overnight affects the quality of your sleep??”
  “I have the same issue; stress causes my levels to rise.”
  “Does anyone else have differences in their Libre adhesive? Some of mine fall off after like 4 days but this last one I had, I pulled the sensor away from the adhesive before the adhesive came off my skin.”
- Relief of blame
  “Very fair! It’s so hard to remember to make changes even as they become routine!”
  “Keep me posted on how it goes! Don’t be mad at yourself for forgetting your things at home...we are human!”
- Other esteem-related support
  “On this day 18 years ago I was diagnosed with T1D. I decided I deserved a (cake emoji).”
  “Happy diaversary! Celebrate today, not the diagnosis but everything you’ve done and accomplished despite the diagnosis.”

**Emotional support (275/3430, 8·02%)**

- Relationship
  “Hahaha aw love you two (tagged names).”
- Physical affection
  “HUGS xx”
- Sympathy
  “Today I hate diabetes...can’t get off this roller coaster. Lows of 1.6, 1.4, 2.1 and 2.9.”
  “Awh man I’m sorry are you switching from Medtronic to a different company? I switched to Tslim recently.”
- Empathy
  “I feel like being a diabetic sometimes holds me back from getting out there and doing something wild because of these worries that run through my head. Ya feel?”
  “I get this all the time too! It scares so much when they say something that diabetes has done to someone they know.”
- Encouragement
  “Wow! That’s a lot of carbs. Flexibility is the best, I’m glad it’s working for you! And I’m sure you’ll figure out the dose :)”
- Other emotional support
  “First time using the Dexcom G6 today! Wish me luck.”
  “The worse part is I constantly have people reminding me about how this virus could kill me because I have a number of medical conditions which messes up my immune system, which stresses me out even more.”

**Network support (100/3430, 2·92%)**

- Access
  “I have a friend who’s t1 and did a 6 month backpacking trip I can send the post to her if you want?”
- Presence
  “If you need to vent and reach out to somebody please message me. I’d rather you reach out than burn out. Not fun!”
- Companionship
  “Hey, i would like to get to know some new people, last person i knew with diabetes sadly passed away a few years ago do to complications...i am in the XXX area. feel free to send a message.”
  “Yes for sure! Shoot me a message when you’re in town.”
- Other network-related support
  “If anyone in the XXX area is available this Saturday to Friday the 24th of August, why not come and work for [Camp name]!!!? [...] If you, or anyone you know is interested please let me know ASAP! Thanks.”
  “Could this short film contest interest some of you? (tagged name) can probably give you more info”

**Tangible support (79/3430, 2·3%)**

- Perform direct task
  “Could you e-mail the prescription to me as well?”
  “I got the whole medtronic transmiting kit for the new pump. Would someone be interested in getting that from me?”
- Active participation
  “HEY HEY VPN friends :) There’s a petition going viral in XXX right now to get pumps covered. GOTTA GET THIS THING SIGNED! Would love if some of you would consider signing.”
  “If you want we could post everyday with one of the challenges here! So everyone can share!”
- Other tangible support
  “I could ship them, and all I would ask is that you reimburse me for the shipping costs. They’re all yours if you want them.”

### Overarching Themes

We identified 10 themes (Table S2 in Multimedia Appendix 2). The first theme, *intersections of T1D with various facets of life*, addressed challenges at school and work that impeded diabetes management; preparation for traveling and moving; and stress related to diabetes, driving, and navigating holidays. The second theme, *relationships between glucose levels and habits*, discussed alcohol, cannabis, and shisha. The third theme, *T1D and social relationships*, focused on family, friends, and romantic partners, with respect to their involvement and support in diabetes management. The fourth theme, *stories about diagnosis, milestones, and identity*, included experiences and emotions at diagnosis, accounts describing personal acceptance of T1D, and preferences related to disclosing the diagnosis to others, with some reporting having tattoos and bracelets related to T1D. The fifth theme, *self-management*, captured goals related to glucose levels, dietary approaches, sleep, and exercise; insulin; and injection and pump sites.

The sixth theme, *stories about meeting and interacting with others with T1D*, was about family members with T1D, local friends and peers with T1D, VPN members, and people at T1D camps and events. The seventh theme, *experiences with health care providers and the health system*, highlighted challenges and transitions. The eighth theme, *T1D and society*, considered ethical, legal, and policy issues, including MedicAlert bracelets, insurance coverage, and blood donation policies. The ninth theme, *discussions about technology, supplies, and materials*, addressed specific issues with meters, sensors, continuous blood glucose monitors, pumps, adhesives, apps, insulin pens, do-it-yourself technological strategies, and troubleshooting. The tenth theme, *T1D and the public sphere*, tackled a range of advocacy activities, including T1D in refugee camps and fundraising. There was a discussion about television characters, athletes, and famous people with T1D, with concerns raised about misrepresentation and stigma.

## Discussion

### Principal Findings

We built a national private Facebook page for persons aged 14 to 24 years with T1D. We recruited and trained peer leaders and provided them with some monetary incentive. Over 33 months, the network grew to include 222 members, with only 11.7% (26/222) of them exiting. No communication was deemed to be inappropriate. Leaders were 10 times more central than regular members, engaging in more posts, comments, and reactions, and having more connections. The density of exchanges peaked at a membership of approximately 50 patients. Views comprised a vast majority of network activities. Over half (3430/6253, 54.9%) of the codes we applied to posts, comments, and polls were social support related, preponderantly in the categories of information sharing and esteem building. VPN-T1D members exchanged information about living with T1D; stories related to key events on the T1D journey; experiences with family, friends, and health care providers; advocacy efforts; and perspectives on gaps in policy. These findings indicate that internet-based chronic disease peer networks such as ours provide social support and that designated and trained peer leaders can be the critical glue within the network. As a network grows beyond 50 members, direct exchanges may decline, but the continued views and membership in our network suggest that the perceived value remains.

### Comparison With Prior Work

As previously noted, SNA was applied to diabetes forums in 3 papers published from 2012 to 2013, 1 by Dias and colleagues [15] and 2 by Chomutare and colleagues [16,17]. In the first paper [15], the researchers applied greedy optimization (Girvan-Newman algorithm and hierarchical agglomeration) preexisting algorithms to an extracted data set of threads and comments from a diabetes forum, to define communities (ie, foci of relatively increased user interaction) in the otherwise vast website landscape. They used centrality computations to identify superusers. In contrast, we applied SNA to 1 closed community. We also identified superusers, which in our case were designated, trained, and incentivized peer leaders. Interestingly, the second paper, conducted by Chomutare and colleagues [16], applied similar methods across 5 forums and discovered that key communities within each of these 5 forums included overlapping leadership. The importance of superusers is reflected in the general 90%-9%-1% principle described across groups of individuals operating in the digital space [30,34]. The 1% of individuals are the superusers who produce the most content.

At the other end are the 90% of individuals who observe without active participation. This is consistent with the fact that 84.9% of the activities in VPN-T1D were classified as views without comment (lurking). Similarly, in a 12-week study of a 43-member mobile health young adult T1D group with a patient researcher moderator in Australia [18], although >60% of the posts were made by the moderator, 87% (n=37) of the members did view each post. A high proportion of lurking was also identified in a very interesting 2020 examination of changes in lurker status within an internet-based diabetes community [28]. The investigators examined the pathways of evolution from lurking to more active participation and vice versa, and their approach offered a sophisticated tool for assessing the impact of efforts to encourage lurkers to engage more actively. However, we would emphasize that viewing without a reaction is nonetheless valued. Those who only view may still gain important information and benefit indirectly from the exchange of empathy and emotional support. In an internet-based questionnaire-based study [35] querying about peer support received through 4 internet-based breast cancer communities, both those who posted and those who viewed the posts experienced similar types of benefits from participation (emotional support, emotional expression, conflict, advice, and insight or universality).

The third SNA paper mentioned earlier, by Chomutare and colleagues [17], evaluated the growth of communities within 2 diabetes forums over time. They divided data by year and determined that the communities within the forums were restructured around new central nodes over time. We divided our data into shorter stable periods, and the designated leaders remained central over the 33 months evaluated. This can be appreciated in our video construction of network growth (Video S2 in Multimedia Appendix 1).

Our analysis revealed similar themes of social support to those seen in other internet-based diabetes communities. For example, in a scoping review of 47 studies examining internet-based organic diabetes communities (Facebook, Twitter [Meta Platforms], Google+ [Google LLC], YouTube [Google LLC], and blogs), social support was most commonly categorized as informational, emotional, or instrumental support or appraisal [36], akin to our categorization of informational, emotional, tangible, and esteem support. Among these studies, the most frequently mentioned topic was shared experiences. Our thematic analysis showed that VPN members frequently shared their personal experiences as a means of peer social support and validation. One major difference between VPN-T1D and some other internet-based diabetes communities is the absence of actual or perceived negative consequences that was readily identified in our study. Because VPN-T1D was monitored by health care professionals, misinformation was uncommon, as it could be quickly corrected or dispelled [37,38]. We did not identify any cases of bullying or aggression either [39]. While it may be unrealistic for all internet-based chronic disease communities to be monitored by health care professionals, groups such as VPN-T1D offer a unique space for both peer support and professional support through information fact-checking. Although our study aimed to characterize the interactions and support mediated in an internet-based peer community, a previous study of a Korean [40] diabetes group demonstrated that perceived computer-mediated social support significantly predicted users’ intentions to actively communicate with their physicians through a sense of empowerment.

VPN-T1D peer leaders were the superusers who represented 4.5% (10/222) of the membership. Similarly, an analysis of posts [30] from 4 UK-based digital health communities related to alcohol, depression, anxiety, and smoking quantified a 74.7%-24%-1.3% user distribution, with the 1.3% of the users comprising the superusers. The authors underscored the importance of encouraging continued superuser engagement for network sustainability. We adopted several strategies in VPN-T1D that likely facilitated this, including peer leader designation, training, and monetary incentives and health professional monitoring and support. None of the regular members demonstrated relative centrality values similar to those of the peer leaders.

A social media content analysis of public Facebook diabetes groups [27] reported that only 8 (24%) of 34 groups designated moderators. Our results suggest that such designation may be helpful. Supporting the possibility that members appreciated the monitoring that we implemented, in a survey-based examination [41] of the use of web-based tools in T1D, 66% of the respondents indicated that they would participate in a group moderated by professionals.

We observed that network density declined as membership numbers increased. The broad social network literature suggests that density and size are interrelated, with more intense communication within smaller groups [42]. An examination of 34 large public diabetes groups (804 to 22,117 members) determined that only 10% of the posts highlighted personal experiences [27]; most sharing was informational. A study of the 15 largest internet-based diabetes groups reported that two-thirds of the posts shared management strategies [25], 13% of the posts were responses to requested feedback, and 29% of the posts were related to emotional support. This was similar to our distribution of exchange types, but in contrast to VPN-T1D, in these groups, 27% of the posts were promotional and related to unproven or unsubstantiated products or services. Somewhat smaller, private, and monitored groups such as ours may promote personal exchanges and reliable information. In our analysis, network density peaked at 50 members, raising the possibility that for some, even a roughly 200-person network may be too large to share personal experiences, let alone public groups with thousands of members.

In a praxiographic-ethnographic study [43], a researcher followed informants with diabetes both within internet-based groups and offline, to understand how they used Facebook to exchange knowledge based on “practical experiments” and “negotiations between bodies, technologies, and daily lives.” Peers cocreated self-care knowledge beyond the biomedical domain, adopting a process of tinkering to create and explore ways of tackling self-care. Communications on VPN-T1D corroborate such tinkering and knowledge cocreation; there was extensive discussion about technology, supplies, and materials.

In VPN-T1D, 60.4% (128/212) of the regular members had female-sounding names, and 70% (7/10) of the peer leaders were female. Some hypothesize that females give and receive more social support [44], and their internet-based language is more emotional support oriented [44]. In general, females are more active on social media than males. We need to determine how best to engage males through internet-based networks to provide them with a social support forum that is more adapted to their needs and communication preferences. For example, among male youths and young adults, video games may provide a communication and connection forum better suited to their habits and preferences [45].

### Limitations

One of the limitations of our study is that we did not query VPN-T1D members regarding their impressions of the network’s value. However, we believe that continued membership and documented activities, including views, indicate the perception of value. We did not assess whether network membership or engagement impacted glycemic control; rather, we focused on the degree and nature of social support within the network and the degree of interconnectedness. A prior study indicated that internet-based communities with a defined emphasis on diabetes management and deliberate involvement of health care providers to this end can lead to improvements in hemoglobin A_1C_ [46]. Our group was on Facebook, but as the social media landscape and demographic preferences change, other platforms may offer alternatives that are equally or more compelling. We captured communications on Facebook but not communications that occurred between members through Messenger or other methods. We did not directly query about sex but instead inferred sex from names, with scope for misclassification. We did not collect or have access to clinical measures such as hemoglobin A_1C_ (a measure of blood sugar control) or BMI, nor did we have information on socioeconomic status (education level or household income).

### Conclusions

In summary, our findings and literature review support the building of internet-based peer communities for T1D and other chronic conditions. These networks provide informational and emotional support in the daily lives of people living with chronic conditions. Superusers are critical to network function. Public health or clinic personnel wishing to build such networks should collaborate with people living with chronic diseases and support peer leaders in becoming superusers. Training, ongoing support, and remuneration of leaders may be important. Internet-based peer communities constitute a growing and valuable resource for chronic disease management. We need continued funding for research, development, and operations to ensure that a wider range of people living with chronic diseases can benefit from such structures within the self-management landscape.

## Appendix

Multimedia Appendix 1 Membership growth through time. Each colored circle represents a node or individual network member (yellow=regular member; blue=designated peer leader; and red=administrator). The line connecting node pairs indicates that a medium to high engagement exchange occurred ≥1 times between the corresponding members. The nodes representing members with more connections are spaced closer together.

Multimedia Appendix 2 Table S1 contains examples of nonsocial support interactions and Table S2 contains overarching themes, and memes.

## Abbreviations

SNA: social network analysis
T1D: type 1 diabetes
VPN: Virtual Peer Network

## Data Availability

The data sets generated during and/or analyzed during this study are not publicly available due to confidentiality considerations as they contain text communications on a private Facebook page (although deidentified). They may be available from the corresponding author on reasonable request.

## References

[1] Punthakee Z, Goldenberg R, Katz P, Diabetes Canada Clinical Practice Guidelines Expert Committee (2018). Definition, classification and diagnosis of diabetes, prediabetes and metabolic syndrome. Can J Diabetes.

[2] Sauder KA, Stafford JM, Mayer-Davis EJ, Jensen ET, Saydah S, Mottl A, Dolan LM, Hamman RF, Lawrence JM, Pihoker C, Marcovina S, D'Agostino RB, Dabelea D, SEARCH for Diabetes in Youth Study Group (2019). Co-occurrence of early diabetes-related complications in adolescents and young adults with type 1 diabetes: an observational cohort study. Lancet Child Adolesc Health.

[3] Harjutsalo V, Pongrac Barlovic D, Groop P (2021). Long-term population-based trends in the incidence of cardiovascular disease in individuals with type 1 diabetes from Finland: a retrospective, nationwide, cohort study. Lancet Diabetes Endocrinol.

[4] Cryer PE (1999). Hypoglycemia is the limiting factor in the management of diabetes. Diabetes Metab Res Rev.

[5] Myers LA, Swanson KM, Glasgow AE, McCoy RG (2022). Management and outcomes of severe hypoglycemia treated by emergency medical services in the U.S. upper Midwest. Diabetes Care.

[6] Leslie RD, Evans-Molina C, Freund-Brown J, Buzzetti R, Dabelea D, Gillespie KM, Goland R, Jones AG, Kacher M, Phillips LS, Rolandsson O, Wardian JL, Dunne JL (2021). Adult-onset type 1 diabetes: current understanding and challenges. Diabetes Care.

[7] Brazeau AS, Nakhla M, Wright M, Henderson M, Panagiotopoulos C, Pacaud D, Kearns P, Rahme E, Da Costa D, Dasgupta K (2018). Stigma and its association with glycemic control and hypoglycemia in adolescents and young adults with type 1 diabetes: cross-sectional study. J Med Internet Res.

[8] Gale EA (2005). Type 1 diabetes in the young: the harvest of sorrow goes on. Diabetologia.

[9] Wu N, Brazeau A, Nakhla M, Chan D, Da Costa D, Mukerji G, Butalia S, Pacaud D, Henderson M, Panagiotopoulos C, Rahme E, Dasgupta K (2020). Type 1 diabetes mellitus virtual patient network as a peer support community: protocol for social network analysis and content analysis. JMIR Res Protoc.

[10] Hilliard ME, Sparling KM, Hitchcock J, Oser TK, Hood KK (2015). The emerging diabetes online community. Curr Diabetes Rev.

[11] Hawe P, Webster C, Shiell A (2004). A glossary of terms for navigating the field of social network analysis. J Epidemiol Community Health.

[12] Christakis NA, Fowler JH (2009). Social network visualization in epidemiology. Nor Epidemiol.

[13] Christakis NA, Fowler JH (2007). The spread of obesity in a large social network over 32 years. N Engl J Med.

[14] Edwards KM, Banyard VL, Waterman EA, Hopfauf SL, Shin H, Simon B, Valente TW (2022). Use of social network analysis to identify popular opinion leaders for a youth-led sexual violence prevention initiative. Violence Against Women.

[15] Dias A, Chomutare T, Botsis T, Mantas J (2012). Exploring the community structure of a diabetes forum. Quality of Life through Quality of Information.

[16] Chomutare T, Arsand E, Fernandez-Luque L, Lauritzen J, Hartvigsen G (2013). Inferring community structure in healthcare forums. An empirical study. Methods Inf Med.

[17] Chomutare T, Årsand E, Hartvigsen G (2012). Temporal community structure patterns in diabetes social networks. Proceedings of the 2012 International Conference on Advances in Social Networks Analysis and Mining.

[18] Ng AH, Crowe TC, Ball K, Rasmussen B (2019). A mHealth support program for Australian young adults with type 1 diabetes: a mixed methods study. Digit Health.

[19] Gavrila V, Garrity A, Hirschfeld E, Edwards B, Lee JM (2019). Peer support through a diabetes social media community. J Diabetes Sci Technol.

[20] Cleal B, Willaing I, Hoybye MT, Thomsen HH (2020). Facebook as a medium for the support and enhancement of ambulatory care for people with diabetes: qualitative realist evaluation of a real-world trial. JMIR Diabetes.

[21] Gabarron E, Larbi D, Dorronzoro E, Hasvold PE, Wynn R, Årsand E (2020). Factors engaging users of diabetes social media channels on Facebook, Twitter, and Instagram: observational study. J Med Internet Res.

[22] Årsand E, Bradway M, Gabarron E (2019). What are diabetes patients versus health care personnel discussing on social media?. J Diabetes Sci Technol.

[23] AlQarni ZA, Yunus F, Househ MS (2016). Health information sharing on Facebook: an exploratory study on diabetes mellitus. J Infect Public Health.

[24] Zhang Y, He D, Sang Y (2013). Facebook as a platform for health information and communication: a case study of a diabetes group. J Med Syst.

[25] Greene JA, Choudhry NK, Kilabuk E, Shrank WH (2011). Online social networking by patients with diabetes: a qualitative evaluation of communication with Facebook. J Gen Intern Med.

[26] Mogi Y, Abedin T, Ahmed S, Gill G, Al Mamun M, Kanda H, Ishikawa Y, Turin TC (2017). Social networking sites for peer-driven health communication: diabetes-related communities in Google. Diabetol Int.

[27] Stellefson M, Paige S, Apperson A, Spratt S (2019). Social media content analysis of public diabetes Facebook groups. J Diabetes Sci Technol.

[28] Kokkodis M, Lappas T, Ransbotham S (2020). From lurkers to workers: predicting voluntary contribution and community welfare. Inf Syst Res.

[29] Sun N, Rau PP, Ma L (2014). Understanding lurkers in online communities: a literature review. Comput Human Behav.

[30] van Mierlo T (2014). The 1% rule in four digital health social networks: an observational study. J Med Internet Res.

[31] Lamb K (2018). Building a network for youth with type 1 diabetes. Diabetes Canada.

[32] Cole-Lewis H, Perotte A, Galica K, Dreyer L, Griffith C, Schwarz M, Yun C, Patrick H, Coa K, Augustson E (2016). Social network behavior and engagement within a smoking cessation Facebook page. J Med Internet Res.

[33] Gaysynsky A, Romansky-Poulin K, Arpadi S (2015). "My YAP family": analysis of a Facebook group for young adults living with HIV. AIDS Behav.

[34] Arthur C (2006). What is the 1% rule?. Guardian.

[35] Setoyama Y, Yamazaki Y, Namayama K (2011). Benefits of peer support in online Japanese breast cancer communities: differences between lurkers and posters. J Med Internet Res.

[36] Litchman ML, Walker HR, Ng AH, Wawrzynski SE, Oser SM, Greenwood DA, Gee PM, Lackey M, Oser TK (2019). State of the science: a scoping review and gap analysis of diabetes online communities. J Diabetes Sci Technol.

[37] Cole J, Watkins C, Kleine D (2016). Health advice from internet discussion forums: how bad is dangerous?. J Med Internet Res.

[38] Hoffman-Goetz L, Donelle L, Thomson MD (2009). Clinical guidelines about diabetes and the accuracy of peer information in an unmoderated online health forum for retired persons. Inform Health Soc Care.

[39] Litchman ML, Rothwell E, Edelman LS (2018). The diabetes online community: older adults supporting self-care through peer health. Patient Educ Couns.

[40] Oh HJ, Lee B (2012). The effect of computer-mediated social support in online communities on patient empowerment and doctor-patient communication. Health Commun.

[41] Giménez-Pérez G, Recasens A, Simó O, Aguas T, Suárez A, Vila M, Castells I (2016). Use of communication technologies by people with type 1 diabetes in the social networking era. A chance for improvement. Prim Care Diabetes.

[42] Friedkin N (1981). The development of structure in random networks: an analysis of the effects of increasing network density on five measures of structure. Soc Netw.

[43] Kingod N (2020). The tinkering m-patient: co-constructing knowledge on how to live with type 1 diabetes through Facebook searching and sharing and offline tinkering with self-care. Health (London).

[44] Tifferet S (2020). Gender differences in social support on social network sites: a meta-analysis. Cyberpsychol Behav Soc Netw.

[45] Lenhart A, Smith A, Anderson M, Duggan M, Perrin A (2015). Teens, technology and friendships. Pew Research Center.

[46] Petrovski G, Zivkovic M (2017). Impact of Facebook on glucose control in type 1 diabetes: a three-year cohort study. JMIR Diabetes.

