# Supplementary material for: Supporting and Incentivizing Peer Leaders for an Internet-Based Private Peer Community for Youths With Type 1 Diabetes: Social Network and Directed Content Analysis
Source: J Med Internet Res. 2023 Dec 12;25:e48267. doi: 10.2196/48267 (PMC10751631; doi:10.2196/48267)

**Supplementary file Appendix: Table 1 *Examples of Non-Social Support interactions***

| **Non-social support** | **45·15% (2823/6253)** |
| --- | --- |
| ***Socializing* (n = 1125)**  “​​HAPPY DIABETES DAY!!! WOOOO”  “Hey Guys they are hosting a great event at my job in Exchange of a donation for [Juvenile Diabetes Research Foundation] enjoy any kind of pizza”  “Welcome to our group!!”  “If there was a group to sponsor refugees with T1D to come to Canada, would anyone be interested in volunteering?”  “It's Stampede time in Calgary and the food they have is insane! How many carbs do y'all think are in deep fried bacon wrapped reese's cups, or cricket grilled cheese?” | |
| ***Conversation Starters/Contributors* (n = 911)**  “hi guys! I was wondering why you find peer support with your diabetes important?”  “I was recently diagnosed in August, and I felt like I had nobody to turn to or learn from. I would rather commiserate with someone who is around my age and understands how upset and frustrated I am, not relate their experiences from 20 years ago to mine today. I also don't know anyone with type 1, only older relatives with type 2 and their symptoms and stories are different from how I live my life and I feel as though in a group like this I am not grouped in with them.”  “Now that the days are getting colder it got me wondering about winter jackets and how the libre still works under them (yay!) But how many layers do you think it would take before it didn't work? Lol”  “Well I went skiing last winter and god I had so many layers and it still worked!” | |
| ***Administrative* (n = 187)**  “We have a new member (tagged name) from Ontario!”  “As the coordinator of VPN, I would like to say THANK YOU for giving the team the privilege to see your thoughts, frustrations and motivations to continue the daily management of #T1D. Our goal was to bring together youth across Canada into a positive support group for #T1D. One of our other goals was to ask YOU what topics the research community should focus on next. Please inspire us by telling us what you want!” | |
| ***Expressions of gratitude* (n =247)**  “Thanks for the comment ill look into that as well with my profs!”  “This is the first I'm hearing of Frio. Thanks!” | |
| ***Banter/Memes* (n=229)**  “You know what grinds my gears? Having to get my prescriptions refilled, like yup I'm still diabetic.”  “Math exam: Bob have 36 candy. He eats 29 of them. What do he got now ? Diabetes. Pardon my english.” “I think we all know that type 1 diabetes isn’t easy peasy at all, but I really do enjoy reading some funny memes about it, even though my non-diabetic friends don’t understand [how] funny they are [laughing emoji] what is your funniest diabetes meme? (See Appendix 3. Memes) | |

**Supplementary file Appendix: Table 2 *Overarching themes***

| **Theme** | **Sub-theme** |
| --- | --- |
| 1. **Intersection of T1D with various facets of life** | |
| **T1D and School/Exams** | |
| “I'm a first year university student and was wondering how diabetic students took their insulin at school? Did the school make accommodations for you or would you just go to the public washrooms to take your doses of insulin […]” | “I haven't found anywhere to do my insulin at my university other than the washrooms to get some privacy. If anyone else has heard about some accommodations I'm interested to hear.” |
| “My lowest bloodsugar was 20mg/dl (1.0mmol) I was in history class and asked to go to the nurse but my teacher refused cause he said I was just saying that to miss class .... back then I was still shy about my diabetes.... so I just sat there till class ended” | |
| **T1D and Working** | |
| “Hello! I was wondering how you all handle diabetes at work. I have my dexcom on my phone, so I would like to be able to have my phone with me. However, I work in a child care centre, and we have really cracked down on having phones out, due to the professionalism side. They are aware I am type one. Normally, I am not sure how to talk to my boss and work about this. If I have to leave the room for a few minutes to do an injection it is not an issue [...] I just worry that when I am looking at my [blood glucose]'s, they will think I am on social media, etc. [...] I know they have to accommodate as it is a medical condition. I just do not know how to have this conversation. Thanks!” | “You have the right to be properly treating and checking your health at your workplace. If you properly communicate your needs to your employer, they need to allow you the time and resources to be able to treat yourself with the care you deserve. I've worked in many daycares and I'm now a teacher. I have my phone on me at all times to check my sugars and I do not leave the room to inject. When you are feeling your best, you can give your best to the kids […]” |
| **T1D and Travel Preparedness** | |
| “Hey guys I’m going to the Philippines for 20 days and I don’t really know how to prepare myself what important things should I know when I’m traveling” | “[…] 1) you'll want to make sure to not overheat your insulin otherwise it won't work too well  2) I usually bring extras in case my bag is lost or something and split it between bags  3) Usually I don't have a problem with airport security but uh, grab a paper from your doctor in case security isn't too cooperative  That's what I can think about [right now]!  Ask if you have any other questions” |
| **T1D and Challenges with Moving** | |
| “So I’m packing up to move and I realize my airport letter from my endo is suuper out of date. Does anyone still use those letters? I’ve never ever been asked for it (and I travel a lot)” | “Mine is out dated too. I still carry it” |
| **T1D and Stress** | |
| “How do you guys deal with midterms? The stress always gets to me and my blood sugars are super sensitive to stress!!  Anyone got tips?  Anyone in the same boat as me?” | “It’s the stress as well as sitting around studying all day (at least in my case). I try to move,bike to uni[versity], be active whenever I can. Good luck”  “I find my control gets a little worse this time of year but I contribute it mostly to exam stress causing me to run high” |

| **T1D and Driving** | |
| --- | --- |
| “Does anyone have any tips about breathalyzers and potentially blowing over because of high [blood glucose]? Just curious. I was always told to say that I’m not refusing to take the breathalyzer but I would rather submit to a blood test if stopped by police [...]” | “My thoughts are that you probably ideally shouldn't be driving if you're ketoney enough to have acetone breath, but just be honest with them and tell them what you know” |
| “For driving, I always make sure I have candy handy. I think that might be a legal issue too” | |
| **T1D and Holidays** | |
| “Hey guys merry Christmas Eve to everyone. With holiday meals fast approaching I want to hear everyone's tips on carb counting, bolusing, if you use a temp basal, etc. Also what is everyone looking forward to this holiday season. Me, I usually give myself a temp basal increase to 150% before we have all our appetizers, for the big dinner I give myself a dual wave Bolus because all the fat and protein in the meal slows the carb down. What does everyone else do?” | “I do the same thing! I calculate as much as possible and try to keep off the heavy carbs because I know I'll be high later no matter what. Drinking LOTS of water helps too!” |
| 1. **Relationships between glucose levels and habits** | |
| **T1D and Alcohol** | |
| “With [New Year’s Eve] tomorrow wanted to share some low carb cocktails, for some ideas! I always find that any carb free drink is easier to control with my diabetes! [link] Comment below any cocktail recipes with carb free/ or any questions you have about drinking and diabetes!”  “When I'm drinking mixed drinks I'll bolus for the first couple then no more - I need some insulin on board but not too much! Hard alcohol has no carbs so it's just the mix to worry about” | |
| **T1D and Cannabis** | |
| “Today is the day Cannabis becomes legal in Canada. Has anyone’s endo been proactive in discussing how it may affect [blood glucose] values? Do you find it affects?” | “The only thing I was ever told was to be aware because of the food cravings that you might not remember to bolus for! Will be interesting to see research for sure.” |
| **T1D and Smoking (Shisha)** | |
| “Does shisha impact [blood glucose]s?” | “I use to smoke shisha quite often and didn't notice any impact !” |
| 1. **T1D and social relationships** | |
| **T1D and Relationships/Partner Involvement** | |
| “Happy Valentine's Day everybody! How involved are your significant others with your diabetes?” | “He knows EVERYTHING” |
| “I wanted to share this with people who are more likely to understand why I'm excited:  The boy I'm seeing just asked if I could recommend some books so that he can get a better understanding of diabetes. My heart is very very happy [heart emoji]. I really really appreciate when people go out of their way to make an effort, especially in a context like this” | |
| **T1D and Involvement of Friends** | |
| “How involved are your friends with your diabetes? Do you have some who try to learn as much as they can? Some who don't want to be engaged?” | “Most of my friends "forget" I have diabetes and will joke about it but they all know if they have any questions to ask”  “My best friend has been involved a lot actually over the years, specifically in the fundraising aspect. I raise money every sprig for [Juvenile Diabetes Research Foundation] and my friend raises money for my team each year. She always attends the walks and kick-off dinners as well which is great [... ] Most of my friends want to learn more, some don't care to, not out of ignorance or anything, just preference and that’s totally okay with me!” |
| **T1D and Involvement of Family** | |
| “So my [blood glucose] was high for several hours today and as I result I felt like crap and still feel like crap so I left work early. My mom is going crazy. She thinks I should of just sucked it up and pushed through. She tried to get me to go back now because my [blood glucose] is fine but I still don't feel great. I just wish she could trust my decision and stop worrying that I will get in trouble at work for taking a sick day once in a while. Any advice???” | “The only advice I could give you is to not let your mom know lol, I have stopped telling my mom what my [blood glucose] is... I'll tell her what my a1c ... but I also will work if my [blood glucose] is high as deal with the issues later” |
| 1. **Stories about diagnosis, milestones, and identity** | |
| **T1D Diagnosis Story** | |
| “Well I was 4 when diagnosed, doctors told me that it was a simple urinary infection and to take antibiotics for 2 weeks and I'd be fine. I went back the next day with severe symptoms and they finally checked my sugar, I was 74.6 (they were surprised I was still alive, let alone walking in by myself), so I got shipped over to the hospital in the town next over where I started my treatment and met my diabetes team. Throughout the years I've had it rough, I was constantly being hospitalized for [diabetic ketoacidosis] (literally almost monthly) doctors kept telling me I shouldnn't still be alive. Fast forward to now, I'm 18 turning 19 soon, haven’t been hospitalized in over a year. And I'm finally on the insulin pump” | |
| **T1D Journey/Milestones/Dia-versary** | |
| “July 10th with be my 19th diaversary. In the past years I have gotten very upset on this day. Just picturing 3 year old me so sick in the hospital. My life forever changed on that day. It also pisses me off that I have had this disease for 19 years! I’m only 22! What is some advice on how to make the day more of a celebration than dreading it and having a really sad day?”  “So it was my diaversary Tuesday and I decided to post some Stories on Instagram about my catheters and my Freestyle [to] do some awareness. Do you do that times to times? I love to do it!” | |
| **Accepting T1D Diagnosis** | |
| “I always say the diabetes diagnosis comes in 2 parts: when the test comes back and you are physically diagnosed, and when you finally accept you have it and learn to incorporate it in your life and make something of it. I was always open about my diabetes, but I really struggled from diabetes burnout and I think only recently (about 15 years after my diagnosis) did I truly accept it. I began to become somewhat okay with having an insulin pump/[continuous glucose monitor] on my body (still hide it with clothes) if I know that it makes me healthier, I started to care more about where my sugars are and what I can do to put them in target.” | |
| **Disclosing T1D/Being Defined by T1D** | |
| “Hey all! When you are told introduce yourself and say three things about you, does diabetes make the list? I used to for my own safety if I missed a low, to explain why I am always carrying food, and because it is unique. More recently, I have dropped saying I am diabetic right off the bat since I don't want diabetes to be the most important thing about me.” | “I almost always tell people right off the bat. Especially at job interviews. They need to know what my health situation is so when I need time/day off for an appointment they understand. But I ALWAYS say I’ve been diabetic for 15 years, but I don’t let it define me. I think if anything it’s made me more resilient and grateful for the life I have” |
| **Coming Across Strangers in the Public who have T1D** | |
| “I was helping a lady find something a work today when she grabbed at her chest and exclaimed "my pump!" This was immediately followed by "sorry, it'll rip out of my stomach otherwise". Inside me was OMG ME TOO but outside me said haha I have one too! It's also in my bra! She asked how long I'd been on a pump, we expressed our enthusiasm for the coming tandem system, we found what she needed, and we went our separate ways. Not a big deal, but meeting [people with diabetes] in the wild is my absolute favourite” | |
| **Tattoos/bracelets** | |
| “[...] any ideas on where to get nice medical jewelry?” | “You can actually get some decently nice ones off of the medic alert Canada website” |
| “I'm looking at getting a tattoo to represent T1D. And I need ideas, let me see yours!! Also any tips, tricks or suggestions for getting the tattoo itself! Thanks” | “[photo of tattoo]” |
| 1. **Self-Management** | |
| **Goals/Motivators** | |
| “[…] What is one thing you want to improve with your diabetes care? […]” | “i would like to improve on my control and bring my a1c below 6 3” |
| “Do you guys have a hands-down least favourite part of diabetes? I think mine has to be nighttime lows...I never had any for five years and now they're so common! My goal is to keep adjusting basals until they're gone forever” | |
| **Managing Blood Glucose Levels** | |
| “When I actually want to care about my bloodsugars but food choices ply a huge impact!”  “I find my blood sugars going low because I’m stressed out and not eating as much food cause I am to focused on studying” | |
| **Hypoglycemia Snacks/Drinks** | |
| “I used to have a small tube of icing sugar in my glucometer kit in case of an unconscious hypo[glycemia] event! They come quite small! Now I just have little tubes of honey cuz it expired.”  “Y'all minute maid has new juiceboxes with no added sugar, meaning each box has 9g of carbs and no gross chemicals :) I loooove juice for lows but I can never find boxes with less than 22g (which is way too much for me) so this is amazing! Plus they're peach mango which is obviously the best kind” | |
| “I was wondering if anyone had any suggestions about low snacks. I get the worst stomach aches when I’m low, and juice boxes just make me feel awful. I'm still so new at this whole diabetes thing” | “Rockets. Halloween is a good time to stock up. For a while I also ate jellybeans. Those I carried in a small pill case. If I am at home, some times I will eat honey straight up when I am tired of rockets. I had a friend who would eat Skittles. (I wonder what % of rockets are eaten to treat diabetes?)” |
| **Preventing Hypoglycemia** | |
| “I just love overcorrecting or over preventing bedtime lows... :/ I cannot wait for basal iQ! Is anyone using tandem and dexcom and can tell me how great it is? [...].” | |
| “[...] I’m training for my first 1/2 marathon so this is all very new to me. But every since I started I’ve been so much prone to getting ketones on long runs [...] by the 12km I felt the ketones. Stopped running and 13km and walked home to test. Any input? How do you prevent the starvation ketones and making sure no [insulin on board] is there when running?” | “Not sure if this helps, I have a recent visit to my nurse and she told me that small ketones are less then 0, Med-Large are between 0.7-1.5 and large is anywhere higher then 1.6. Also to go to the hospital if over 3.0” |
| **Hyperglycemia** | |
| “[...] I feel like everytime I eat my [blood glucose] spikes uncontrolably and no matter what I correct it just won't come down past 12-13mmol _ been feeling really run down by the constant highs these days idk whats going on! Suggestions anyone? [...]” | “I definitely recommend prebolusing! I'll sometimes go as early as 20 minutes if I'm high” |
| **Carb Counting** | |
| “Quiz time (brought to you by my latest horrifying grocery shopping experience) :) : If Craisins Original have 33g per serving, and Craisins Reduced Sugar claim to have 50% less sugar, how many carbs do Craisins Reduced Sugar actually have?”  “I stay low carb most of the time! And I try to measure my food to help with carb counting. But I also test a often after big meals just in case I messed up!”  “Check out this site for carb counting wowow [link]” | |
| **Sleep** | |
| “Anyone else feel that having high blood sugar overnight affects the quality of your sleep??” | “ABSOLUTELY I was very high most of last night and woke up very groggy & feeling physically exhausted” |
| **Weight Loss** | |
| “so im super interested in T1D and fat loss... so i was wondering if anyone here or if u guys know anyone who has T1D and have tried to lose weight. how was the experience? did you guys do it through exercise, nutrition or a combo? [...] how were your blood sugars? did u guys see any fat loss results” | “now I’ve been trying to lose weight but nothing serious. My biggest recommendation is avoid the quick fix diets it will have to be a combination of eating healthy and exercise. [...] Definitely book an appt with a dietician too...they can be a handy source” |
| “I also don't have a dietician. [...] If I did, however, I'd love to ask him/her for healthy diet meal plans to help lose weight as a T1D!” | |
| **Listening to Body** | |
| “I'm wondering if anyone has found they've lost their ability to feel highs as effectively as you could before? I used to be able to feel them at around 16 mmol/L but in the last couple months, unless it’s first thing in the morning I have climbed to numbers of even 20 without feeling the usual symptoms... my A1C is below 7 and has been for years, so it's not like I'm running high all the time and have become desensitized... not sure what's going on or if anyone can relate!” | “All I know is it can happen if you've had a period of high days. Might not reflect on your a1c but if you've had a period...say 1-2 weeks of highs. You might feel them less often. I'm that way with lows. I don't feel them until i’m at 2.5 but I can feel my highs at 11”  “I feel my highs often. Especialllly when I wake up. What are your usual symptoms that you look for?” |
| **Insulin** | |
| “Kills me. I could take 200% more insulin overnight and it doesn't appear to do anything (my basals are already as high as 1.2units). Sometimes if I go to bed at 6 it'll stay at 6...and sometimes it'll go up to 12 all on its own. [...]” | |
| **Climate Impacts** | |
| “ [...] I am currently in Peru and the altitude is extremely high in some places. My blood sugar levels are very difficult to manage since I arrived here and I am often in hyper for no reason and my sugar levels don't go down despite several corrections. My freestyle sensor stopped working at about 5000m altitude. In addition, sickness altitude symptoms resemble a lot like ketoacidosis symptoms so it is difficult to differentiate them. Anyways, I advise you to acclimatize gradually to the altitude and bring a looooooooot of diabetes care supply.” | “I have heard of altitude impacting blood sugars but have never personally experienced it! Thank you for sharing, I'm sorry it's going like this for you!”  “I've done my fair share of mountains and high altitudes and I've never had an issue!” |
| “I had frozen insulin before so now I keep any insulin on my in insulated pack. If i’m going for a hike or winter activities I keep my glucometer in my inside pocket of my coat so it's close to body temp” | |
| **Injection/pump sites on body** | |
| “Being Ms. Incredible for Halloween and trying to think where to put my omni pod to not let it show.... Pump users do you find this being an issue???[...]” | “I don’t do my sites on my lower back! I’m too scared so I usually stick to my leg tummy and arm but recently my arms have been awful absorption sites” |
| “Can anyone tell me why none of my regular site spots are comfortable anymore? I use Medtronic Mio infusion sets (the straight-in kind, not the 45 degree ones) [...]Recently I've been gaining muscle and continuing to use my stomach, butt & arms regularly as site spots, but they always start to hurt (they feel like bruises) after 1-2 days? Any advice or techniques to get a full 3 days in without having to rip a brand new site out? Thanks!” | “Try a different kind of site? Make sure there is no scar tissues or lumps” |
| **Logbooking** | |
| “Does anyone track their [blood glucose] using an app, excel, book, etc? I use excel so I can have more control over what I track- like averages for example.” | “I use My Sugr logbook app! It's amazing and predicts A1C levels, automatically makes graphs, and has options to include notes, [insulin on board], trends, all that. I recommend it to everyone!!” |
| “I struggle with laziness. [...] I am terrible at recording my readings in a logbook. It sometimes feels like I've put my health on the back burner when it should be my #1 priority.” | |
| **Mental Health / Emotions** | |
| “[...] Diabetes plays a huge role in our mental health too and it's important to keep everything balance. Mental health is as important as our physical health! We're all doing great”  “[…] I believe it would be good idea to offer one appointment yearly (in addition to our 3 month follow ups) that focuses not solely on our numbers, but on mental health. I find it really refreshing to have conversations about diabetes that don't surrounding numbers. Young adulthood is a very stressful and busy time in life; it would be nice to sit down and just have a conversation about some of these stressors (good or bad).” | |
| **Support system** | |
| “[...] I think that having a good support system, and having peers with T1D, would help. It helps hold yourself and your peers accountable, and you want to succeed for yourself and for them”  “[...] I was diagnosed at te age of 8 and was lucky enough to right away get a great support system: my mom, my doctor, Camp [name] we're all great for a young kid not very sure what it meant to be diabetic. [...]”  “It's me, my dad and my brother haha (all type 1) I didn't really think about it growing up, but I've never been concerned about my diabetes because my dad knew that going low or high wasn't the end of the world. I find this different from a lot of parents who (of course) get more worried about that stuff because they don't understand it on the same level. Now that I'm older I realize how lucky I am to have that support, someone to ask questions, complain to, and someone to reassure me that what I'm doing IS good enough” | |
| **Alternative/Herbal Treatments** | |
| “I have a lot of family like that, the grandpa I live with has a natural remedies book that he tries to convince me to use to cure my diabetes. There was also an Ojibway tribe chief that tried telling me that if I drank a tea brewed from the cattails found by creeks that are going up stream I’lll never have to check my sugar again plus I work in an ice cream shop and have random people telling me these things all the time so I just got used to saying yeah I think I'll try that sometime”  “We all laugh at the ridiculous cure suggestions people offer...but I'm reading an article about the use of natural health products in diabetes management and it turns out that cinnamon actually can lower blood sugar! However, it doesn't affect A1c and has no effect on controlled diabetes. So really it's nothing, but I guess those suggestions do have a foundation!” | |
| **T1D Friendly Recipes** | |
| “I've been struggling with foods in the morning that don't spike my sugars cause dawn phenomenon hits me hard. These waffles have been a life saver. I added lily chocolate chip to mine and a little but of sugar free vanilla skinny syrup. 2g net carb without the chocolate. 8g with”  “With [New Year’s Eve] tomorrow wanted to share some low carb cocktails, for some ideas! I always find that any carb free drink is easier to control with my diabetes! [link] Comment below any cocktail recipes with carb free/ or any questions you have about drinking and diabetes!” | |
| **T1D and Menses/illness/Surgery** | |
| “Anyone have experience with wisdom teeth surgery and type 1? They want to [intravenously] sedate me which means I’ll be totally unconscious. And I forgot to ask about how they’re going to deal with the diabetes bit” | “[...] The risk is in case you throw up and then can’t breathe. So if you do lose control, eat the minimum you can and then talk to the anesthesiologist. You may not want to eat after (your mouth will hurt) so maybe have some sugary fluids on hand in case you go low after the surgery. I also didn't take enough pain meds so I was an unhappy camper recovering at home. So my advice would take the maximum dose of drugs they let you take. I’d also recommend having a parent or friend who know diabetes to help you test after the surgery and keep an eye on you at home. My surgery was pretty short too.” |
| “oo yes I find like 4-5 days before my period insulin is of no help when I’m high... then when I get my period it’s all lows”  “do you guys find your blood is higher or lower when you have the stomach flu? It knocked me out this past weekend and I that’s keep my blothat’s. Wasnt –ating mthat’s Maybe thats why? but normally being sick makes me run higher. any thoughts?” | |
| **Vitamin D** | |
| “Do any of you supplement Vit[amin] D? I know [type 1]s are usually deficient but I grew up in a sunny place and always played outside so never really worried about vit[amin] d until recently because I was feeling fatigued all the time and turns out my blood work said 33 when the normal is 75-125. If you do supplement, are there any specific brands you like? There are many kinds and strengths on the market” | “I take 1,000U Vitamin D daily in the winter plus a Multivitamin. In the summer I don't since there is more sun and I'm often outside. I have never been formally tested or diagnosed for a Vitamin D deficiency as far as I know.” |
| **Footcare** | |
| “How's everyone’s feet? Do you do anything special to take care of them? As it’s a new sandal season, I’m covered in blisters and they take FOREVER to heal.” | “Keep them clean!! The blisters I mean lol” |
| **T1D and Exercise** | |
| “Just got a gym membership. I need all the tips and tricks on how to avoid going low!” | “Depends a lot on what type of activity you'll be doing at the gym! Long-duration, low intensity, cardio exercise will tend to make you go low, while in some people weight lifting and high-intensity activities can actually spike your [blood glucose]. Any idea on what kind of workouts you'll be doing?” |
| “[...] what do you do when you exercise? Do you adjust your bolus, basal, the amount you eat? Do you keep your pump on or take it off? This year i started wearing my pump during hockey and i noticed by blood was a lot better. and I wasnt chasing highs all day after practice. I also only ever play with my bolus or have an extra snack. and I test before, during and after cause im paranoid like that ahahah” | “I make sure I'm above 8 before I start, remove my pump during, and then bolus as soon as I'm done no matter what I'm at. But I do a lot of rollercoastering. I relate!” |
| **T1D and Diets (Keto, Gluten-Free, Plant-Based)** | |
| “Hey guys! I was wondering your thoughts on keto? Let me know where you fit and why please!! Options: not interested, thinking about getting on keto, currently on keto, have done keto in the past” | “I think it all depends on the reason, duration and kinds of foods you eat in keto” |
| “What did you discuss with your dietitian the last time you saw him/her?” | “I see a dietitian every time, in the past they haven't been much help but in this past year I have switched diets to a vegan and gluten free. My dietician has been very open to trying to find good protein and a balanced diet.” |
| 1. **Stories about meeting and interacting with others with T1D, outside VPN-T1D** | |
| **Family Member(s) with T1D** | |
| “curious to know if you guys have anyone with T1D in your family? Mom, ,dad, grandparent, aunt, uncle, cousin, sibling? no one?” | “My mother and my sister are both type 1!” |
| “It's funny because my dad, who has T1D wasn’t the one who noticed. But my mom remembered how much water my dad had drank and made the connection with [me]” | |
| **Peers with T1D** | |
| “A friend of mine used their thigh, like on the side where you'd inject, and she said it's accurate!”  “Definitely try and distract myself with anything other than diabetes related stuff. I always try and remember of all the fellow type 1s who I've encountered along the way who are going through the exact same thing as me. It kind of puts it in to perspective, even if you're having a bad day, we've all been there and it will get better.” | |
| **T1D Camps/Events/Retreats** | |
| “[...] I was diagnosed at 8 months old, and started attending the d[iabetes] camps program when i was seven, and I've been back every summer since either as a participant or staff, it has heavily influenced who i am. Diabetes has become pretty important in deciding who I've become. Its offered me some incredibly unique awesome and fun opportunities as well as letting me meet some pretty awesome people, all in all diabetes maybe isn't the greatest but its definitely added some positive things to my life” | |
| “Hi everyone! Who went to a diabetes summer camp? I did! How did you like your experience?” | “I went from 11-13 years old and it was an amazing experience. It was so much fun and it was also really comforting, it helped me feel like I fit in and it was great to get to know people that understood what I was going through. I’m still friends with a few people I met at camp today!” |
| **Memes** | |
| 1. **Experiences with health care providers and the health system** | |
| **Impact of Physician's Communication** | |
| “I know I'm quite lucky that my adult endo is T1D and is willing to answer emails to make up for a lack of seeing each other. I've heard terrible stories of my friends not seeing an endo for years because they can't find an appointment, OR because they detest going so much that they just avoid it :( I think we need adult endocrinologists who focus on T1D and recognize that treatment is unique to each person, unlike a lot of other diseases. Criticism/hurtful comments, whether intentional or not, is NOT the way to encourage better care/alleviate burnout, and using a one-size-fits-all approach is unacceptable” | |
| **Diabetes Care Team** | |
| “I hated my transition, mostly because I had a fantastic team in peds who I loved and then I...didn't anymore. There is a nurse team here who have been helpful but they are still quite [type 2] focused (although it's getting better). They did have a transition clinic where I got to see the nurse/dietician/doctor but I only got to go twice as my endo (endocrinologist) decided I didn't need it anymore. I hated my adult endo, I didn't feel she cared or listened. I told her I was anxious and burnt out and her response was "yeah, you're at high risk for that". I finally switched endos, I met my new one yesterday and I love her! (I encourage you all to endo shop if you're not enamored).” | |
| **Transition from Pediatric Care to Adult Care** | |
| “How many of you went through transition from peds to adults? If you have what was good or bad about your transition? If you haven’t been what are somethings you’d want to know/happen before or during the transition process? I’m presenting about transition to a whole bunch of endos and educators and i’d really appreciate your input” | “Incredibly difficult! Finding an endo has been almost impossible for me and I’ve received 0 support or assistance from my old healthcare team. It's gotten to the point where it may affect my [Assistive Devices Program] coverage!! The system is incredibly flawed”  “In my ideal adult healthcare world, it would still be a clinic approach, appointments would be every 3 months instead of every 6, and the first meeting would include the peds team as well as the adult endo. Also it would make my life if they had in-clinic a1c machines like they do in peds because having to go to the lab separate from my appointment kills me. [...]” |
| **Challenges with Adult Endocrinologist** | |
| “yeah tbh I’ve been so busy with school that I haven't bothered since my management is good right now I don't feel the extreme need. I have found a diabetes educator that I like. Eventually I will.”  “my last appt was so frustrating but just like you i'm like nah too much of a hassle to switch...I only see him every 8 months anyways”  “[...] I am a full time student and haven't seen him in over a year because I can never make it to his appointment times (he only works Mon-Thurs most weeks and I go to school out of province). He lets me email him with questions but I miss having face-to-face conversations about my struggles. He means well but he's also got a 3-month wait list for bookings so I really wish I was prioritized more, sometimes I feel a bit more like a patient number than a human” | |
| 1. **T1D and Society** | |
| **Ethical/Legal Concerns** | |
| “[link to article titled “diabetic teen dies after prescribed oils instead of insulin - the herbalist is going to prison”] Reading through the post the man barely got 4 months in jail, such a malicious spread of misinformation that lead to this poor boys death, how is this justice? I would like to see more effort in educating people on what diabetes is and what will actually help them. No reason this should have happened.” | “ [...] I also wonder if the parents should be charged...this is a horrible situation and my heart hurts so much for that boy. I am so angry that we have people like this in society who choose to ignore medical science and talk other people (like vulnerable kid's parents) into doing crap like this. I am also super fired up 100% of the time about anti vaccination stuff and this is not dissimilar... [...]”  “I would consider it as murdering someone so the charge should be equal. Cause it's a situation that could've been avoided” |
| “A few days ago, a Victoria, [British Columbia] teen riding the public bus was feeling hypo[glycemic]. She was about to eat a snack when the bus driver told her not to because of the "no food" rule on the buses. She explained that she could have a "life-threatening seizure if she didn't eat the snack to raise her blood sugar back up. [...]" | |
| **Institutional Restrictions for Members Living with T1D** | |
| “Hey, I am part of the national cadet programme. Because of diabetes they have given me a bunch of restrictions that need to be followed. One of them is that all diabetic cadets need to be at a maximum of 30 minutes from a hospital. Has anyone ever been told to never go further then 30 min from a hospital? Personally me I live in the middle of no where and it's pretty where that when I travel I stay close to 30 minutes from a hospital.” | “I was also in cadets and I did have that set as a limit though it would've been impossible for me to go to any functions like camp or corps trips. My family and I sat down with the commanding officer of my corps and actually set out a plan that was to be followed if we were more than 30 minutes away. It was really a lot of self advocation for something that ( I found) was unfair.” |
| **Emergency Alert Systems (i.e. Medic Alert)** | |
| “Fair enough! When I started with medicalert we had to pay extra for them to keep health info on file...looking at it now though I think you might be able to just get the bracelet”  “Not to scare you ... but after a girl passed out in one of my lectures and no one knew anything about her medical history...I realized if I was to pass out without wearing a medical bracelet no one would know I have diabetes” | |
| **Careers in T1D** | |
| “I worked at D-camps as a student nurse this summer! It was exciting and inspiring and even though I struggled a little bit it made me realize that I maybe really do want to work with [type 1] kids in my career. Now I'm getting more involved in [I Challenge Diabetes] and I can't wait to see what the future holds!”  “[...] I am thankful for diabetes. For better or for worse, it has made me who I am. It has pushed me to pursue my dreams in the face of fear (because if im in charge of keeping myself alive every day, then it doesn't get much scarier than that). It has led me to what I hope will be a fantastic and fulfilling career, and it has led me to meet amazing people and be part of incredible communities that I never would have had access to otherwise.[...]” | |
| **T1D and blood donation** | |
| “Can diabetics donate blood?” | “They told me that it’s because removing a large volume of blood can cause dangerous fluctuations in our sugars”  “ In Quebec, [type 1]s can't donate blood because they are using insulin every day. As we don't want to give blood to people who aren't diabetics and we don't want to provoke hypoglycemia episodes, we cannot donate blood. It's an exclusion criteria of Hema-Quebec.” |
| 1. **Discussions about technology, supplies, and materials** | |
| **Glucometers** | |
| “Currently I am trying to decide if I should switch to the free style libre from a regular metre.”  “Thoughts on rechargeable vs battery powered devices? I'm loving my new rechargeable meter. But the new pump I want is rechargeable and I'm really anxious about that with no certainty as to why. Any insight or ideas? How do you keep everything working?” | |
| **CGM** | |
| “There are some pumps on the market (at least in the US) that have closed loop insulin delivery where a pump changes the basal output based on [continuous glucose monitor] readings. I think this is super cool and could improve my A1C. This seems pretty new to me so maybe there is some ongoing research about how this technology, user interaction could be improved, or what some of the broader effects are.”  “I hate having something attached to me 24/7 and I only use a pump! My endo/parents keep pushing for me to use a pump and a [continuous glucose monitor] but I think I'd feel so limited with that many sites on me. I want to go back to injections but I have way less control” | |
| **Pump (pump breaks)** | |
| “I want to hear all of your opinions on different pumps! My warranty is up soon so I might change pumps. I have the old Medtronic pump right now. I will never use a sensor (enlite or dex com) as I use the freestyle libre.” | “I’ve heard great things about the new Medtronic pump. The only reason I’m not switching to it after my Animas warranty is because I do happen to use Dexcom, so I’m waiting for Tandem (rumour has it end of this year release)’ |
| “[...] I've been taking a pump break for the last couple weeks (I usually do in the summer just for a breather and because I'm home all the time, reliable schedule, and it's pretty chill) and I am absolutely LOVING it. In the past I have always gone back on my pump for school, but I kinda don't want to this time. However, I have always been a strong supporter of pumping and have always thought of it as the best possible care. So I'm a little weirded out that I kinda want to ditch it. I'm also scared because there are some features you lose when on pens. Should I give it a go anyway? [...]” | “I am also enjoying a pump break right now and considering being off the pump more long-term. I think if you get similar blood sugar results on pens or pump, you can choose what fits best with your preferences and lifestyle. We have to deal with this disease every day so we may as well have some choice in how we manage it. You always have the option to switch back to the pump at some point.” |
| “Hey to my fellow insulin pump & tights wearing people: How do you wear your pump? Tucked into your tights? In a sweater pocket? Any super cool pump accessories you’ve found to help with this? I'm so sick of always being worried it’s gonna fall down my leg - not the comfort level I’m going for! Help!” | |
| **Pump Rental Programs** | |
| “[British Columbia] friends on pumps: I'm wondering how funding works for British Columbia residents. I plan to move west when I finish school and in Ontario I receive pump funding through ADP (assistive devices program) via the government. I basically have to provide A1C results annually as well as have my endo sign off saying I test regularly, know what to do if I have ketones and see an endo regularly. Does [British Columbia] have something similar?” | |
| **Adhesives/sensor attachment/skin reactions** | |
| “I recently switched from a dexcom to a Libre, and I had a couple questions. What is the best tape/adhesive to keep it on? Additionally, the rise/fall rate, how much do you rise/fall by? Any other tips and tricks would be greatly appreciated” | “I always used skin tac and hypafix, not super necessary during the winter, but safe. I actually just switched from the libre to dexcom and i hate it so far. Its constantly losing signal/malfunctioning and hurts i find.” |
| “[...] on Saturday I put in a fresh Libre sensor right before going out (rookie mistake!) and when I got home the adhesive was starting to lift off of one side. SO, after scouring my room, I ended up using false eyelash glue to stick it back down, and it worked perfectly!”  “I applied a few drops onto my skin, right under where the sensor was starting to lift, and then pressed it down until it dried. I've taken many showers since then and it's held up just like other, more expensive adhesives!”  “I also find I don't break out or get a rash from it, opposed to other adhesives. And it stays on in the shower, but it is easy enough I don't need anything to help me get it off.” | |
| **Technological Advances** | |
| “There are some pumps on the market (at least in the US) that have closed loop insulin delivery where a pump changes the basal output based on [continuous glucose monitor] readings. I think this is super cool and could improve my A1C. This seems pretty new to me so maybe there is some ongoing research about how this technology, user interaction could be improved, or what some of the broader effects are.”  “MiniMed 670G has been approved for Canada. The news reports say that you may be able to buy it as sson as December. What is cool about this pump is that it has a [continuous glucose monitor] feedback loop to adjust insulin. I am so excited!” | |
| **Technological Burnout** | |
| “Does anyone have any experience using the medtronic 670 pump? I'm at my wits end with omnipod.. every other pod I put on ends up leaking and my sugars spike up to 20. I've tried troubleshooting everything but nothing I do seems to help! The only thing holding me back from switching is I've seen looping.. it's been so good for my a1c… thanks in advance”  “While I appreciate the technological advances that these companies have helped with, I do worry about it becoming more of a consumerist state. iPhones cost upwards of $1000 despite costing pennies to make...is the same going to happen to [continuous glucose monitors]s? And, if non-diabetic people start seeing their daily blood sugar trends, I think we are going to create a very anxious population (as well as one that wonders even more loudly why type 1s can't just diet)” | |
| **Troubleshooting (Temperature)** | |
| “Omni Pod users: Do you cover your pod if you will be outside in the sun for a long period of time??[...] ” | “I don't cover it when I'm in the sun. I'm usually outside for work all day and this past week has been very warm. I don't find that my pump gets warm from the sun [...]” |
| “[...] Those of you in colder parts of the country, do you have to do anything special when temperatures drop? I know my glucometer wouldn't work in the cold once...anyone ever had a frozen omnipod?” | “I had frozen insulin before so now I keep any insulin on my in insulated pack. If i’m going for a hike or winter activities I keep my glucometer in my inside pocket of my coat so it's close to body temp” |
| **T1D Finances/Insurance - Access/Cost of Supplies/Insulin** | |
| “I have a question, if you don't have any insurance are there any ways to get coverage for sensors? I'm getting really bad with checking my [blood glucose] levels and I want to get sensors really bad, but they cost so much!”  “[...] There's a petition going viral in Manitoba right now to get pumps covered. GOTTA GET THIS THING SIGNED! Would love if some of you would consider signing”  “[...] This November, one of my friends (from D-Camp, shoutout) was a huge advocate for pump funding - WHICH STILL DOESN'T EXIST IN MANITOBA! I've had two pumps in my life - one that my parents purchased, and one that was raised via crowd funding (GoFundMe). We all know that T1D is an expensive illness, but the coverage is SO varied across Canada. What's coverage like where you life? What supplies/technology do you pay for? If any?” | |
| “Hey y'all Do any of you guys worry about insurance coverage in the future?? So scary that laws and coverage differ so much from place to place and seems like there's never any consistency” | “I hate that it's something we have to consider when looking for jobs”  “[Ontario Health Insurance Program]+ now covers insulin, test strips, lancets for everyone under 25, just need to show your health card and prescription to the pharmacist” |
| “I've been thinking about this a lot lately as I finish school in April and that means I lose my parents' amazing insurance plan. Also means I need to get a real job... Is anyone out there on their own yet? How is the insurance thing going? Anything you wish you knew or wish you did differently?” | “I wish I knew not all jobs have insurance LOL I'm paying $378 a month just for insurance that only covers the cost of insulin and hospital stays” |
| “Hi! I'm looking at going back on a pump, and currently, I am leaning towards t-slim. However, I just learned that it is not covered by the Alberta pump program. My endo told me (if I remember correctly) that many peoples private insurance will cover it. Or will cover the cost of supplies, but not the pump cost? Can anyone advise, and what is your coverage like?” | “I'm in the same situation, but in [British Columbia] :) Find someone to contact at your insurance company - most will have an online contact form. That's the only way to find out for sure. The plan I'm on right now will cover $4000 for a pump every 5 years and covers supplies at 80% to a max of $3000. I haven't heard of any companies that don't cover supplies, but I know a lot of plans have specific restrictions for pumps” |
| **Waste and recycling** | |
| “[...] are there any diabetes supplies “garbage” that can be recycled? I feel terrible contributing to so much waste but I know I can’t give up my supplies (obviously). Alternatively, what management programs produce the least amount of waste? (Pumps vs. [multiple daily injectios] for example)” | “Pods have a recyclable waste program” |
| “[...] I'm having some issues with the new biohazard containers that pharmacies are distributing. I use the mio infusion sets and they a) don’t fit in the containers and b) fill them in like 2 weeks. Is there is different way to get rid of the infusion sets or am I stuck? Let me know!” | “I pull the needles out of my sites (with pliers) and put the needles in the biohazard bin, then blue-box recycle the plastic parts! It's definitely doable, the only thing I actually throw in the garbage are strips (and they sometimes go in the hazard bin, depends)” |
| **Diabetes Applications** | |
| “The G6 most probably won't be available in Canada until next autumn, but good to know: its app has cool features such as access to Clarity (the software with all your data) directly from the app.”  “I just discovered this new app called sugarmate. It connects to your Dexcom and has a bunch of neat features. I recommend trying it out” | |
| **Customer service and technology** | |
| “Do you guys think if I call Medtronic and complain, I can get a new enlite transmitter even though my warranty is done?”  “My brand new dex[com] sensor is throwing question marks already...how soon should I call them? Anyone had much experience with dexcom customer service?” | |
| **Insulin pens** | |
| “I finally tracked down a NovoPen Echo (1/2 unit insulin pen), thanks to those of you who suggested asking my clinic. Is anyone else using it?”  “thanks I might do that. For now I got Flextouch pens but I think since I am sensitive to insulin the 1/2 unit pen would be better.” | |
| **DIY (Do it yourself)** | |
| “DIY: sometimes my tudes hold well but recently my days have been spent in the water with kids, and they unstick a bit on the 2nd or 3rd day, and the needle starts to hurt me because it moves inside. So, having only dexcom stickers, I cut it them haha”  “Any comments on whether people like the [DIY] better than the Medtronic 670?” | |
| **Imaging, scans and technology** | |
| “[...] Good news, it’s not diabetes related, bad news, I’m still hospitalized. I’ll probably have to do various x-rays and scans, have any of you guys done scans with your pump or [continuous glucose monitor]? [...]” | “I had an [magnetic resonance imaging] done not that long ago and they say you're supposed to take off sensors because they can fry in the machine and burn your skin” |
| “[...] I would just give it to the security people and ask them to swab it. I don't think the sensor causes issues but don't go through the full-body scanner” | |
| 1. **T1D in the Public Sphere** | |
| **T1D Refugee Programs** | |
| “If there was a group to sponsor refugees with T1D to come to Canada, would anyone be interested in volunteering?” | “So I have been personally involved with refugee sponsorship through Canada's private sponsorship program. In this program, a group of community members comes together, chooses a refugee family overseas that they want to sponsor, fundraises to support them for a year, and then welcomes them to Canada and helps them adjust for their first year in Canada. [...] The hope would be to bring some refugees with T1D to Canada so they can access quality medical care and supports, because we know that with the right supports it is possible to live a great life despite T1D.” |
| **T1D Education - General Public** | |
| “I just taught two Grade 11 Chemistry classes about Diabetes, and all that. Pretty much what we learned when we were first diagnosed, condensed into a 50 minute teaching session” | |
| **Raising T1D Awareness** | |
| “So it was my diaversary Tuesday and I decided to post some Stories on Instagram about my catheters and my Freestyle yo do some awareness [...]”  “For my diaversary I will be making the diabetes ribbon shaped sugar cookies to take to work with me to thank my co-workers and managers for all the support they offer [...] but I also get a few comments from some co-workers about my diabetes so I wanna leave like a nice info graphic or something about what is diabetes and some info about t1” | |
| **Patient Groups & Fundraising** | |
| “Hey everyone! I'm doing a charity garage sale fundraiser for [group name]. Last year we raised $1200 and our goal this year is $2500! I know we can achieve it and with all of our community’s help, it will be awesome! I’m accepting monetary donations, beer cans and bottles and old items that you don't want anymore. [...] Thanks for all of your love and support, hope to see you all at some point that day.”  “To raise awareness for National Diabetes Month I have teamed up with [Juvenile Diabetes Research Foundation] Canada and made a video series all about busting those T1D myths!! This is the first video where I mimic a Vogue 72 questions video! Comment below your biggest T1D myths” | |
| **Athletes with T1D** | |
| “I look up to Max Domi: he plays for the Coyotes in the NHL. The guy is super tough and has so much skill! He also wears a medtronic pump so im a fan! [...]” | |
| **Famous people with T1D** | |
| “Do any of you have any T1D idols or people that inspire you?? Mine has got to be Nick Jonas... before giving my needles as a kid I honestly used to say "well if Nick can do this while touring, and performing, than a 10 year old can definitely do this!" [...]” | |
| **Books and podcasts** | |
| “I’m not sure if you’re into podcasts but PancreasPals has an episode on dating with Diabetes!! My boyfriend got right into it, he’ll really help me manage it and is usually the one reminding me to bolus or helping me carb count. [...]!”  “Diabetes books! I know this has been a topic on here before but I thought I would add some of my opinions. I wanted nothing to do with diabetes books until very recently. [...] Here are a few of my faves! [Book Name] - [Author Name]. I love this book. Her blog (blog name) is fun, real-life, and super inspiring. Her book is much the same! It's all anecdotal about real life with diabetes and definitely helped me to take a deep breath and relax [...] I hated the thought of diabetes reading for so long but I've found it really helpful and a really good way to find out that I'm not along in feeling how I feel!” | |
| **TV characters with T1D** | |
| “Do you guys know of any TV shows, movies, or books where one of the characters has T1D?” | “When I first watched go figure i’m like omg she’s just like me”  “There's an episode in the walking dead series that features a diabetic survivor” |
| **Stigma** | |
| “I get so annoyed when people always associate diabetes with sugar and being overweight! It shouldn’t bother me but for some reason it really does. It creates a stigma around diabetes in a whole. Not clarifying type 1 or type 2 and when people brush off jokes like this saying we’re politically butthurt, when they have no idea what it feels like. The ignorance sometimes gets me so mad. How do you guys feel seeing posts with people comparing sugar/unhealthiness with diabetes and making fun of it not knowing the facts?”  “A few days ago, a Victoria, [British Columbia] teen riding the public bus was feeling hypo[gylcemic]. She was about to eat a snack when the bus driver told her not to because of the "no food" rule on the buses. She explained that she could have a "life-threatening seizure if she didn't eat the snack to raise her blood sugar back up." The bus driver still did not let her eat. The teen was upset at the driver's response and feels that he should have made an exception because this was a medical situation. Click here to listen to what Dr. Kaberi Dasgupta said about this incident: the public should be made aware of what #type1diabetes is and there is stigma surrounding T1D [link]” | |

**Supplementary file Appendix : Memes**


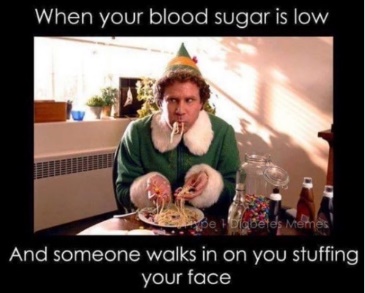

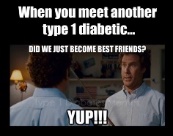

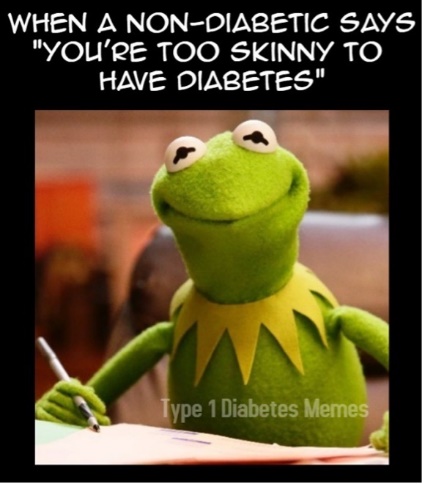

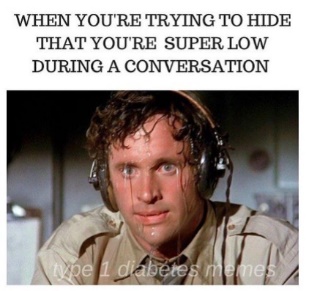

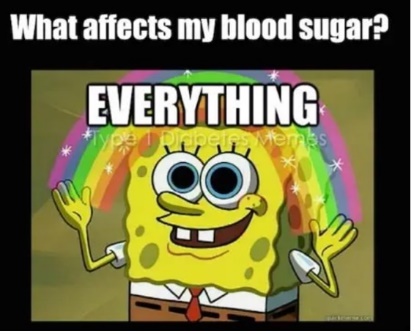

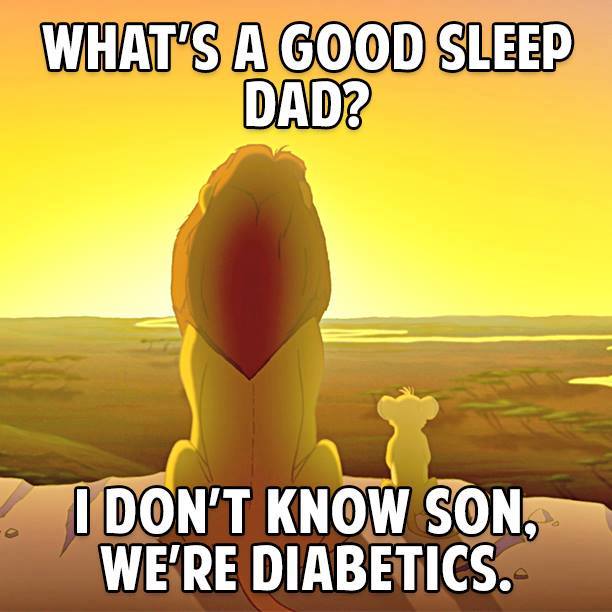

Supplement: Multimedia Appendix 2 [file jmir_v25i1e48267_app2.docx]
